# Integrative Approach to Reveal Cell Type Specificity and Gene Candidates for Psoriatic Arthritis Outside the MHC

**DOI:** 10.3389/fgene.2019.00304

**Published:** 2019-04-11

**Authors:** Matthew T. Patrick, Philip E. Stuart, Kalpana Raja, Sunyi Chi, Zhi He, John J. Voorhees, Trilokraj Tejasvi, Johann E. Gudjonsson, J. Michelle Kahlenberg, Vinod Chandran, Proton Rahman, Dafna D. Gladman, Rajan P. Nair, James T. Elder, Lam C. Tsoi

**Affiliations:** ^1^Department of Dermatology, University of Michigan Medical School, Ann Arbor, MI, United States; ^2^Morgridge Institute for Research, Madison, WI, United States; ^3^Department of Biostatistics, Center for Statistical Genetics, University of Michigan, Ann Arbor, MI, United States; ^4^Ann Arbor Veterans Affairs Hospital, Ann Arbor, MI, United States; ^5^Division of Rheumatology, Department of Internal Medicine, University of Michigan, Ann Arbor, MI, United States; ^6^Division of Rheumatology, Department of Medicine, University of Toronto, Toronto, ON, Canada; ^7^Centre for Prognosis Studies in the Rheumatic Diseases, Krembil Research Institute, University of Toronto, Toronto, ON, Canada; ^8^Institute of Medical Science, University of Toronto, Toronto, ON, Canada; ^9^Department of Laboratory Medicine and Pathobiology, University of Toronto, Toronto, ON, Canada; ^10^Faculty of Medicine, Memorial University of Newfoundland, St. John’s, NL, Canada; ^11^Department of Computational Medicine and Bioinformatics, University of Michigan, Ann Arbor, MI, United States

**Keywords:** psoriatic arthritis, gene candidates, systems biology, epigenomics, GWAS

## Abstract

We recently conducted a large association analysis to compare the genetic profiles between patients with psoriatic arthritis (PsA) and cutaneous-only psoriasis (PsC). Despite including over 7,000 genotyped patients, only the MHC achieved genome-wide significance. In this study, we hypothesized that appropriate epigenomic elements (H3K27ac marks for active enhancers) can guide us to reveal valuable information about the loci with suggestive evidence of association. Our aim is to investigate these loci and explore how they may lead to the development of PsA. We evaluated this potential by investigating the genes connected with these loci from the perspective of pharmacogenomics and gene expression. We illustrated that markers with suggestive evidence of association outside the MHC region are enriched in H3K27ac marks for osteoblast and chondrogenic differentiated cells; using pharmacogenomics resources, we showed that genes near these markers are targeted by existing drugs used to treat psoriatic arthritis. Significantly, six of the ten suggestive significant loci overlapping the regulatory elements encompass genes differentially expressed (FDR < 5%) in differentiated osteoblasts, including genes participating in the Wnt signaling such as *RUNX1*, *FUT8*, and *CTNNAL1*. Our approach shows that epigenomic information can be used as cost-effective approach to enhance the inferences for GWAS results, especially in situations when few genome-wide significant loci are available. Our results also point the way to more directed investigations comparing the genetics of PsA and PsC.

## Introduction

Genome-wide association studies (GWAS) are a well-developed hypothesis-free approach for identifying susceptibility loci of complex diseases, and have been applied to study different skin conditions (Tsoi et al., 2018). For instance, many distinct genome-wide significant loci have been revealed for psoriasis among Caucasian patients (Tsoi et al., 2017; Patrick et al., 2018), and the information gained from these studies can be used to improve our understanding of disease pathogenesis as well as to guide further experiments. Nevertheless, there are two main limitations: first, disease-associated loci are often positioned in non-coding regions, and it is not trivial to reveal their cell-type specific gene targets; second, many loci remain undetected, due to their subtle effect sizes (Boyle et al., 2017). Increasing the sample size does help to enhance statistical power and reveal more loci (Jansen et al., 2018; Mahajan et al., 2018), but this can be difficult (and expensive) to achieve for diseases with relatively low prevalence, such as psoriatic arthritis [<0.5% (Alamanos et al., 2008)]. Therefore, integrative approaches are necessary to decipher the biological implications of GWAS results when genome-wide significant loci are unavailable.

Typical GWAS approaches ignore potentially valuable information by only reporting markers showing genome-wide significant association. We and others have illustrated that epigenetic and pharmacological data can be used to provide biological inference for GWAS (Okada et al., 2014; Farh et al., 2015; Tsoi et al., 2017; Patrick et al., 2018). Recently, we performed a GWAS meta-analysis of psoriatic arthritis (PsA) vs. cutaneous-only psoriasis (PsC), using over 7,000 psoriatic patients across 6 cohorts (Patrick et al., 2018). Even though PsA has been estimated as having over 80% heritability (Greb et al., 2016), no markers outside the major histocompatibility complex (MHC) were genome-wide significant; however, we showed that markers outside the MHC are also informative to provide PsA assessment among psoriatic patients. In this study, we utilize an integrated approach to analyze the biological mechanisms involving suggestive significant loci. By leveraging additional independent information, we identify potential disease-relevant genes from the suggestive significant loci outside the MHC and provide enhanced interpretation of further pathological mechanisms for psoriatic arthritis. Our work illustrates that valuable information is embedded in association results for loci which fall shy of genome-wide significance, and our integrated approach can be used to extract that information from GWAS results.

## Methods

### PsA vs. PsC Meta-Analysis

**FIGURE 1:**
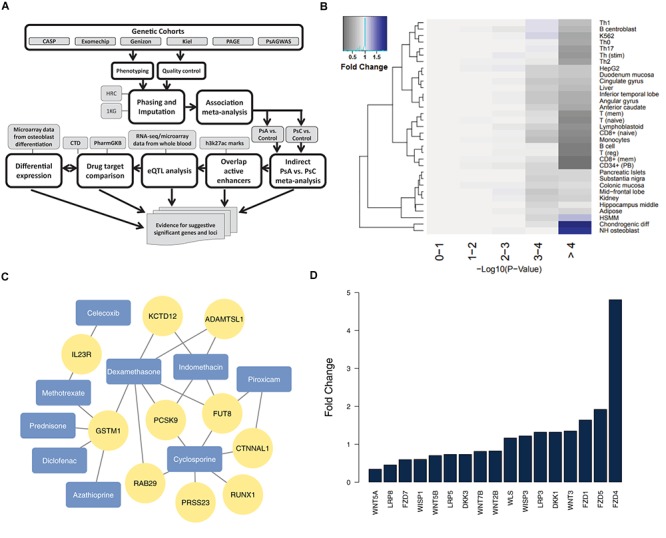
Analysis of suggestive significant PsA vs. PsC loci outside the MHC **(A)** our pipeline for identifying and analyzing non-MHC suggestive significant loci; **(B)** H3K27ac marks for active enhancers reveal chondrogenic and osteoblast cell type enrichment (negative logarithmic *p-value* shown) among suggestive significant (*p* < 1 × 10^-4^) markers – fold change is determined by comparing the proportion of markers that overlap active enhancers for a particular cell type in each –log10 *p-value* with that of the 0–1 reference bin; **(C)** genes from suggestive association loci are targets of drugs for PsA (arthritis drugs from DrugBank are colored in blue, and their drug targets from CTD/PharmGKB in yellow); **(D)** Wnt signaling genes are differentially expressed (FDR < = 5%) in differentiated osteoblasts.

The overview of our workflow is illustrated in Figure 1A. We utilized previous meta-analysis results (Patrick et al., 2018), consisting of 3,566 dermatologist-diagnosed PsC and 3,674 rheumatologist-diagnosed PsA patients of Caucasian descent from 5 GWAS datasets: CASP (Nair et al., 2009), Exomechip (Tsoi et al., 2017), Genizon (Ellinghaus et al., 2010), Kiel (Ellinghaus et al., 2010), and PsA GWAS (Stuart et al., 2015) and one Immunochip dataset PAGE (Tsoi et al., 2012). We defined PsC patients as having psoriasis for ≥10 years without a PsA diagnosis. Informed consent was received from all patients in accordance with the Declaration of Helsinki and the protocols approved by each institutional review board. Quality control, phasing/imputation (using HRC/G1K reference panels) and association analysis were performed as described previously (Patrick et al., 2018). Meta-analysis (Willer et al., 2010) was performed separately for PsA vs. control and PsC vs. control, and we compared the summary statistics results with a chi-square statistic for indirect meta-analysis (Stuart et al., 2015).

### Prioritization of Candidate Cell Types and Genes

Our previous study illustrated that the most significant markers in the PsA vs. PsC association (mostly in the MHC) were enriched in regulatory elements in immune cells (Patrick et al., 2018). Here, our study focused on association results outside the MHC (26–34 Mb of chromosome 6), and we overlapped the suggestive significant (*p* < 1 × 10^-4^) markers identified by indirect PsA vs. PsC meta-analysis against active regulatory elements, measured by H3K27ac marks (Farh et al., 2015). The enrichment of cell types with active enhancer overlap was evaluated at different levels of significance in PsA vs. PsC meta-analysis, compared to markers which overlap active enhancers available for any cell/tissue type. Arthritis drugs were identified from DrugBank (Wishart et al., 2018) and their gene targets extracted from the Comparative Toxicogenomics Database (CTD) (Davis et al., 2015) and PharmGKB (Thorn et al., 2013), which were merged together in our previous work (Raja et al., 2017). We identified the most significant eQTL targets, for the markers overlapping the regulatory elements, from a study with whole blood from 31,684 individuals (Võsa et al., 2018), to identify the most probable genes for each locus. Differential expression analysis was conducted using moderated t-statistics from a linear model fitted by limma (Ritchie et al., 2015) in GEO2R (Barrett et al., 2013) on a previous study (van de Peppel et al., 2017) which characterized the gene expression of human osteoblast cells differentiation from mesenchymal stromal cells: we compared pre-differentiation versus 3-day post-differentiation from three replicated samples, used the most significant probeset when multiple were available, and defined significant differentially expressed genes (DEGs) as yielding False Discovery Rate (FDR) <5%.

## Results

### Enrichment of Suggestive Significant Markers Among Active Enhancers for Normal Human (NH) Osteoblasts and Chondrogenic Differentiated Cells

We overlapped all 165 suggestive significant (*p* < 1 × 10^-4^) PsA vs. PsC markers (Supplementary Table 1) from 49 distinct loci outside the MHC (loci being defined as contiguous regions encompassing multiple markers, with <500 kb genomic distance between any two suggestive significant markers) against H3K27ac chromatin marks. There are 53 markers (18 loci) which overlapped active enhancers for any cell type; we used these in Fisher’s exact test as the baseline reference for enrichment of specific cell types: we compared the number of suggestive significant/non-significant markers which overlap active enhancers for a specific cell type, with those that overlap active enhancers for any other cell type (Supplementary Table 2). Chondrogenic differentiated cells (*p* = 3.99 × 10^-5^, OR = 3.1, Fold Change (FC) = 1.9; 30 markers from 10 loci) and osteoblasts (*p* = 3.33 × 10^-4^, OR = 2.7, FC = 1.8; 29 markers from 7 loci) are the only cell types showing significant enrichment (after Bonferroni correction). Indeed, the suggestive significant markers tend to have context-specific overlap with chondrogenic cells and osteoblasts (with 1.91 and 1.75 times more overlap compared to baseline significance, respectively), as they show decreased overlap for most other cell types (Figure 1B).

### Genes Near the Suggestive Significant Loci Which Overlap Active Enhancers for Osteoblast and Chondrogenic Cells Are Targeted by Drugs Used to Treat Psoriatic Arthritis

Cartilage and bone destruction/growth is known to play a significant role in the development of PsA, and potential biomarkers have been identified that are involved in their regulation (Chandran et al., 2010). By focusing on the suggestive significant markers that overlap active enhancers for the enriched cell types, we aimed to identify associated gene targets by utilizing drug-gene interactions (Okada et al., 2014; Tsoi et al., 2017). We used our compiled drug-target database to identify target genes nearby for each of the 10 loci that overlap active enhancers for osteoblast or chondrogenic cells (Table 1 and Figure 1C), and found the diseases these drugs are used to treat. The genes were identified using eQTLs or distance to each locus; the drugs that target these genes were identified using the CTD/PharmGKB database, from which we selected drugs used to treat arthritis according to DrugBank. We included the most plausible gene from every locus (using eQTL or distance) as well as the drugs that target these genes. Significantly, all 10 loci encompass genes targeted by at least one PsA drug. Four of the drugs (celecoxib, diclofenac, indomethacin, and piroxicam) are non-steroidal anti-inflammatory drugs (NSAIDs), but there are also three immunosuppressive drugs (azathioprine, cyclosporine, and methotrexate) and two corticosteroids (dexamethasone and prednisone), used to treat psoriasis in general. Furthermore, three of the most significant loci (encompassing *RUNX1 p* = 2.95 × 10^-5^, *FUT8* *p* = 4.44 × 10^-5^ and *CTNNAL1 p* = 1.02 × 10^-5^, respectively) are thought to be involved in Wnt signaling (a key regulator of bone formation) (Merdek et al., 2004; Kurimoto et al., 2014; Chimge et al., 2016).

**Table 1:** Suggestive significant (*p* < 1 × 10^-4^) loci overlapping active enhancers for osteoblast or chondrogenic cells, ranked by GWAS *p-value* of most significant marker (min *p-value*) overlapping regulatory elements for either cell type.

Locus	Osteoblast min p-value	Chondrogenic min p-value	Notable nearby gene	Drug with target in the locus
11q14.2 (85993121–87015072)	2.27 × 10^-5^ (rs7123349)	1.52 × 10^-5^ (rs7940637)	*PRSS23* (eQTL: *p* = 1.21 × 10^-7^; rs1939110)	Cyclosporine
21q22.1 (36472001–37559234)	4.91 × 10^-5^ (rs2835060)	2.95 × 10^-5^ (rs2242761)	*RUNX1* (proximity: intron; rs2242761)	Cyclosporine
14q23.3(65202594–66220065)	4.44 × 10^-5^ (rs7146907)	4.44 × 10^-5^ (rs7146907)	*FUT8* (eQTL: *p* = 1.30 × 10^-19^; rs7146907)	Cyclosporine, Dexamethasone, Indomethacin, Piroxicam
9q31.2(110560196–111560196)	4.95 × 10^-5^ (rs77922938)	4.95 × 10^-5^ (rs77922938)	*CTNNAL1*(eQTL: *p* = 1.02 × 10^-5^; rs77922938)	Cyclosporine, Piroxicam
9p22.2 (17928324–18941918)	5.08 × 10^-5^ (rs9406750)	8.91 × 10^-5^ (rs7043305)	*ADAMTSL1* (proximity: 32 kb; rs7043305)	Dexamethasone, Indomethacin
1p31.3 (67103343–68144173)	NA	5.92 × 10^-5^ (rs7539795)	*IL23R* (eQTL: *p* = 1.03 × 10^-24^; rs7539795)	Celecoxib, Methotrexate
1p13.3 (109829214–110833792)	8.64 × 10^-5^ (rs139201738)	7.54 × 10^-5^ (rs6698967)	*GSTM1* (eQTL: *p* = 1.29 × 10^-5^; rs6698967)	Azathioprine, Dexamethasone, Diclofenac, Methotrexate, Prednisone
13q22.3 (76955805–77955805)	7.63 × 10^-5^ (rs373098847)	7.63 × 10^-5^ (rs373098847)	*KCTD12* (proximity: 3’ UTR; rs373098847)	Dexamethasone, Indomethacin
1q32.1 (205107153–206107153)	NA	7.70 × 10^-5^ (rs77244682)	*RAB29* (eQTL: *p* = 3.14 × 10^-11^; rs77244682)	Cyclosporine, Dexamethasone
1p32.3 (55018752–56018752)	NA	8.05 × 10^-5^ (rs7552841)	*PCSK9* (proximity: intron; rs7552841)	Cyclosporine, Dexamethasone, Indomethacin


*For the markers we include in the eQTL column, we first selected from the suggestive significant (PsA vs. PsC) markers that are eQTLs in the database, and overlapped them with the regulatory elements. If multiple markers satisfy the criteria, we selected the most significant marker-gene association for presentation and, where no markers satisfy the criteria, we provide the proximity to the nearest gene, along with the drug targets for each gene. For example, while rs1939110 is not the most significant marker overlapping active enhancers for osteoblast (rs7123349: *r*^*2*^ = 0.72) or chondrogenic cells (rs7940637: *r*^*2*^ = 0.60) in the first locus, it is suggestive significant for PsA vs. PsC (6.47 × 10^-*5*^) and it is the most significant eQTL (*p* = 1.21 × 10^-*7*^).*

### Differential Expression

We then investigated their differential expression following osteoblast differentiation. Interestingly, 6 of the top 10 loci shown in Table 1 encompass genes significantly differentially expressed (FDR < 5%) in differentiated cells: *PRSS23* (FC = 1.29, *p* = 4.89 × 10^-3^, 11q14.2), *RUNX1* (FC = 0.658, *p* = 1.30 × 10^-3^, 21q22.12), *FUT8* (FC = 0.706, *p* = 3.70 × 10^-3^, 14q23.3), *CTNNAL1* (FC = 0.633, *p* = 1.34 × 10^-5^, 9q31.2), *ADAMTSL1* (FC = 0.757, *p* = 3.35 × 10^-6^, 9p22.2), *GSTM1* (FC = 1.49, *p* = 1.56 × 10^-4^, 1p13.3); from loci with suggestive evidence of association that do not overlap with osteoblasts/chondrogenic differentiated cells, we also observed differentially expressed genes that participate in the Wnt signaling pathway: *WNT5A* (FC = 0.344, *p* = 3.24 × 10^-6^, 3p14.3), *DKK1* (FC = 1.32, *p* = 2.06 × 10^-6^, 10q21.1) and *FZD4* (FC = 4.81, *p* = 7.11 × 10^-10^, 11q14.2) (Figure 1D). Differential regulation of genes during differentiation, as well as genes involved in the same pathway (e.g., Wnt signaling) provide further evidence of genuine signals among the suggestive significant loci identified by GWAS.

## Discussion

When working with suggestive significant loci, there is always a danger of false positives. Independent epigenomic information allows us to focus on loci that have an effect on cells and tissues relevant to the disease, and thus are more likely to represent genuine signals. Focusing on suggestive significant loci that overlap active enhancers for NH osteoblasts and chondrogenic differentiated cells, we were able to identify genes that are targets of PsA drugs and differentially expressed following osteoblast differentiation. A suggestive significance threshold of *p* < 1 × 10^-4^ was chosen, as it is sufficiently stringent to reveal differential cell type enrichment (Figure 1B) while still allowing 165 markers to meet this threshold. The results when taken together implicate the potential Wnt signaling involvement in PsA development (Goldring and Goldring, 2007; Gudjonsson et al., 2010). Our approach makes it possible to go beyond the information available from genome-wide significant loci to identify testable mechanisms by which suggestive significant loci may impact the development of disease.

Dividing genetic markers into distinct loci, from which to identify gene candidates, can be challenging due to their overlapping genetic position. To address this, we performed cell type enrichment analysis on the marker rather than locus level (Figure 1B), then divided markers into loci according to their genetic distance. To evaluate the impact linkage disequilibrium (LD) might have on these results, we clustered the 165 suggestive significant markers according to their LD, estimated using PLINK 1.9 (Chang et al., 2015) on samples of European ancestry from the 1000 Genomes Project (The 1000 Genomes Project Consortium, Auton et al., 2015) by applying Ward’s criteria (Murtagh and Legendre, 2014) for hierarchical clustering (ward.D2 in R’s hclust package) with an *r*^2^ threshold of 0.9. Comparing the enrichment of cell types in these clusters against randomly sampled loci, chondrogenic cells (*p* = 5.0 × 10^-4^) and osteoblasts (*p* = 3.6 × 10^-2^) again indicated enrichment.

Existing functional annotations provide further information for candidate gene prioritization: genes from the top loci (*RUNX1*, *FUT8*, and *CTNNAL1*) are known to be involved in Wnt signaling; and in 11q14.2, *PRSS23* is a target of a *cis-*eQTL (*p* = 1.22 × 10^-7^) in whole blood (Võsa et al., 2018), and another gene in this locus (*FZD4*) was the most significantly upregulated gene (FC = 4.81) in osteoblast differentiated cells. An eQTL for *FZD4* is available for a marker in LD with the locus (*r*^2^ = 0.76) in T-cells from umbilical cord (Dimas et al., 2009), but there is no direct *cis-*eQTL in whole blood. Among arthritis drugs, *PRSS23* is only targeted by cyclosporine, whereas *FZD4* is also targeted by dexamethasone, diclofenac, and indomethacin. However, a recent study using crisprQTL mapping (Gasperini et al., 2018) found the median distance between enhancers and their target gene to be 34.3 kb (*PRSS23* is 5 kb away, whereas *FZD4* is 160 kb away), so further work would be needed to assess the true causal gene in this case.

Another gene in the same locus as *CTNNAL1* is *KLF4*, which has been found to interact with TNF-α in rheumatoid arthritis (Choi et al., 2018). However, unlike *CTNNAL1* (FC = 0.633), *KLF4* is not significantly differentially expressed in osteoblasts, and the suggestive significant markers in this locus do not overlap any active enhancer marks for T-cells (in which TNF-α is produced). Similarly, *AMPD2* is in the same locus as *GSTM1* (157 kb vs. 80 kb from the lead marker) and it is a gene target for the biologic drug tofacitinib, yet it is not differentially expressed in differentiated osteoblasts. When identifying genes from (genome-wide or suggestive significant) loci to design follow-up experiments to understand the potential biological mechanisms, it is important to consider all the information available.

Biologic drugs are increasingly being prescribed for psoriatic arthritis and it is worth mentioning that (with possible exception of tofacitinib for *AMPD2*) none of the drugs targeting the genes we identify are biologics. The loci (and hence the genes) we present were selected according to their overlap of H3K27ac marks for osteoblasts/chondrocytes, due to their enrichment among suggestive significant markers, and biologics (e.g., anti-TNF/IL23) target the immune system with high precision. It is important to note drugs mentioned in this paper are not necessarily more effective for PsA and further (preferably *in vitro*) studies would be necessary to evaluate the differential effect for drugs in skin and joints. Instead, we identified the drugs that target each gene as an independent means to verify the plausibility of those genes acting in mechanisms which differentiate PsA from PsC.

Epigenomic information has previously been used to suggest causal variants for autoimmune diseases (Farh et al., 2015) and cancer (Han et al., 2015). Recently, an approach was proposed to identify pairs of potentially interacting variants, in active enhancers and gene promoters, respectively (Manduchi et al., 2018). These approaches make it possible to prioritize genetic markers and genes, but it can be difficult (in the absence of further experiments) to know whether the suggested markers and genes are correct. Our study goes further by investigating available pharmacogenomic and expression data to provide independent information available through epigenomics and suggest potential ways in which the loci identified may be involved together in biological mechanisms for pathogenesis.

## Links for Databases

DrugBank: https://www.drugbank.ca/ (March 2019).
Comparative Toxicogenomics Database: http://ctdbase.org/.
PharmGKB: https://www.pharmgkb.org/ (the March 2016 version was used in this paper).
eQTL database: http://eqtlgen.org/.
Data on osteoblast differentiation: https://www.ncbi.nlm.nih.gov/geo/query/acc.cgi?acc=GSE80614.

## Author Contributions

LT designed the study and directed the analysis. JE coordinated and led the psoriasis genetic cohorts. MP conducted the analysis, and LT, KR, SC, and ZH provided bioinformatics or statistical analysis support. JV, TT, JG, JK, VC, PR, DG, RN, LT, and JE provided biological inferences or interpretation of the results, or contributed to the collection/data coordination of the samples in the cohorts. MP and LT wrote the first draft of the manuscript, and every author has reviewed the work.

## Conflict of Interest Statement

JG serves as Advisory Board for Novartis, AbbVie and MiRagen, and has received research support from AbbVie, SunPharma, and Genentech. JK serves on an advisory board for AstraZeneca. The remaining authors declare that the research was conducted in the absence of any commercial or financial relationships that could be construed as a potential conflict of interest.
