# Supplementary material for: Integrative Approach to Reveal Cell Type Specificity and Gene Candidates for Psoriatic Arthritis Outside the MHC
Source: Front Genet. 2019 Apr 11;10:304. doi: 10.3389/fgene.2019.00304 (PMC6470186; doi:10.3389/fgene.2019.00304)
Supplement: Supplementary file 1 [file Data_Sheet_1.docx]

## **Supplementary Tables**

**Supplementary Table 1: Suggestive Significant Markers Outside the MHC**

| **Marker ID** | **Chr** | **Position (hg19)** | **Alleles (risk/nonrisk)** | **PsA AF** | **PsC AF** | **Meta OR** | **Meta SE** | **Meta p-value** |
| --- | --- | --- | --- | --- | --- | --- | --- | --- |
| rs34673906 | 1 | 3457419 | A/G | 0.973 | 0.963 | 1.55 | 0.105 | 3.07x10^-5^ |
| rs145303718 | 1 | 6917187 | A/AT | 0.162 | 0.129 | 1.20 | 0.046 | 5.78x10^-5^ |
| rs56029510 | 1 | 6955905 | G/GT | 0.161 | 0.129 | 1.21 | 0.046 | 5.73x10^-5^ |
| rs55820697 | 1 | 6955913 | C/G | 0.161 | 0.129 | 1.21 | 0.046 | 5.70x10^-5^ |
| rs5772471 | 1 | 11774979 | G/GC | 0.902 | 0.879 | 1.26 | 0.058 | 8.87x10^-5^ |
| rs7552841 | 1 | 55518752 | C/T | 0.398 | 0.386 | 1.09 | 0.022 | 8.05x10^-5^ |
| rs6588242 | 1 | 67603343 | C/T | 0.463 | 0.423 | 1.09 | 0.021 | 6.37x10^-5^ |
| rs6588243 | 1 | 67603383 | C/A | 0.463 | 0.423 | 1.09 | 0.021 | 6.37x10^-5^ |
| rs1158199 | 1 | 67606139 | C/T | 0.509 | 0.464 | 1.09 | 0.021 | 5.07x10^-5^ |
| rs7539795 | 1 | 67609446 | G/A | 0.509 | 0.465 | 1.09 | 0.021 | 5.92x10^-5^ |
| rs7520667 | 1 | 67610639 | A/C | 0.509 | 0.465 | 1.09 | 0.021 | 5.74x10^-5^ |
| rs6588245 | 1 | 67611799 | T/C | 0.509 | 0.464 | 1.09 | 0.021 | 5.74x10^-5^ |
| rs6588246 | 1 | 67612216 | C/T | 0.509 | 0.464 | 1.09 | 0.021 | 5.86x10^-5^ |
| rs2902439 | 1 | 67617346 | C/G | 0.506 | 0.463 | 1.09 | 0.021 | 7.48x10^-5^ |
| rs2863204 | 1 | 67617756 | G/A | 0.506 | 0.463 | 1.09 | 0.021 | 7.40x10^-5^ |
| rs2180295 | 1 | 67622192 | T/G | 0.506 | 0.463 | 1.09 | 0.021 | 7.04x10^-5^ |
| rs12755265 | 1 | 67622814 | A/G | 0.506 | 0.463 | 1.09 | 0.021 | 7.34x10^-5^ |
| rs143977358 | 1 | 67627292 | CTA/C | 0.501 | 0.453 | 1.09 | 0.021 | 1.79x10^-5^ |
| rs7532161 | 1 | 67642223 | G/A | 0.470 | 0.428 | 1.09 | 0.021 | 8.75x10^-5^ |
| rs6660812 | 1 | 67644173 | C/G | 0.470 | 0.428 | 1.08 | 0.021 | 9.67x10^-5^ |
| rs55785704 | 1 | 110329214 | T/C | 0.034 | 0.017 | 1.49 | 0.102 | 9.41x10^-5^ |
| rs60775830 | 1 | 110329538 | A/G | 0.034 | 0.017 | 1.50 | 0.102 | 7.20x10^-5^ |
| rs6691948 | 1 | 110329555 | A/G | 0.034 | 0.018 | 1.50 | 0.102 | 7.70x10^-5^ |
| rs12087417 | 1 | 110329628 | C/G | 0.035 | 0.018 | 1.50 | 0.102 | 8.11x10^-5^ |
| rs6687356 | 1 | 110329674 | C/G | 0.034 | 0.017 | 1.50 | 0.102 | 7.42x10^-5^ |
| rs6698967 | 1 | 110331728 | A/G | 0.036 | 0.020 | 1.47 | 0.097 | 7.54x10^-5^ |
| rs139201738 | 1 | 110333792 | G/T | 0.024 | 0.012 | 1.64 | 0.127 | 8.64x10^-5^ |
| rs77244682 | 1 | 205607153 | G/A | 0.030 | 0.019 | 1.37 | 0.080 | 7.70x10^-5^ |
| rs142050201 | 2 | 59916935 | A/C | 0.990 | 0.970 | 2.74 | 0.234 | 1.54x10^-5^ |
| rs193298986 | 2 | 161521081 | G/A | 0.016 | 0.009 | 1.61 | 0.121 | 8.03x10^-5^ |
| rs35623257 | 2 | 182450966 | T/G | 0.134 | 0.111 | 1.16 | 0.037 | 5.87x10^-5^ |
| rs776622081 | 2 | 182484053 | CT/CTT | 0.145 | 0.120 | 1.18 | 0.039 | 1.73x10^-5^ |
| rs146800461 | 3 | 978500 | G/A | 0.991 | 0.983 | 1.67 | 0.125 | 4.06x10^-5^ |
| rs1200269641 | 3 | 8690249 | G/GC | 0.985 | 0.976 | 1.65 | 0.121 | 3.62x10^-5^ |
| rs9854484 | 3 | 67189354 | C/T | 0.482 | 0.442 | 1.10 | 0.025 | 8.97x10^-5^ |
| rs12488315 | 3 | 67211884 | C/T | 0.495 | 0.453 | 1.09 | 0.021 | 8.60x10^-5^ |
| rs9825439 | 3 | 67271303 | C/T | 0.411 | 0.365 | 1.12 | 0.026 | 7.31x10^-6^ |
| rs13324987 | 3 | 103788557 | C/T | 0.039 | 0.028 | 1.31 | 0.068 | 6.73x10^-5^ |
| rs13079191 | 3 | 103810091 | C/T | 0.035 | 0.024 | 1.33 | 0.073 | 8.48x10^-5^ |
| rs71325557 | 3 | 103810624 | T/C | 0.036 | 0.025 | 1.33 | 0.073 | 8.41x10^-5^ |
| rs114210080 | 3 | 156756843 | T/A | 0.055 | 0.039 | 1.30 | 0.065 | 4.65x10^-5^ |
| rs149361019 | 3 | 156782075 | G/C | 0.053 | 0.037 | 1.31 | 0.067 | 6.45x10^-5^ |
| rs62409254 | 4 | 25425838 | T/A | 0.078 | 0.061 | 1.22 | 0.051 | 8.18x10^-5^ |
| rs77590734 | 4 | 73558588 | G/A | 0.055 | 0.037 | 1.41 | 0.083 | 3.79x10^-5^ |
| rs144834866 | 4 | 73559170 | C/T | 0.056 | 0.038 | 1.39 | 0.082 | 5.28x10^-5^ |
| rs12499612 | 4 | 73559822 | T/C | 0.055 | 0.037 | 1.40 | 0.082 | 4.13x10^-5^ |
| rs12510216 | 4 | 73559988 | C/T | 0.055 | 0.037 | 1.40 | 0.082 | 4.15x10^-5^ |
| rs189603166 | 4 | 73560387 | T/G | 0.055 | 0.037 | 1.40 | 0.082 | 3.94x10^-5^ |
| rs143211589 | 4 | 73560629 | C/T | 0.055 | 0.037 | 1.40 | 0.082 | 3.96x10^-5^ |
| rs147528993 | 4 | 73560710 | G/A | 0.055 | 0.037 | 1.40 | 0.082 | 4.02x10^-5^ |
| rs34477661 | 4 | 100387950 | TAA/TA | 0.914 | 0.893 | 1.21 | 0.044 | 2.53x10^-5^ |
| rs72683244 | 4 | 155762272 | T/C | 0.988 | 0.980 | 1.51 | 0.106 | 9.04x10^-5^ |
| rs116193323 | 5 | 16969407 | A/G | 0.043 | 0.005 | 4.34 | 0.373 | 8.32x10^-5^ |
| rs143150324 | 5 | 107654074 | TA/T | 0.857 | 0.824 | 1.17 | 0.035 | 6.29x10^-6^ |
| rs77145280 | 6 | 88904444 | T/C | 0.973 | 0.962 | 1.32 | 0.071 | 7.76x10^-5^ |
| rs3735290 | 7 | 5873245 | C/T | 0.081 | 0.065 | 1.20 | 0.047 | 9.90x10^-5^ |
| rs311395 | 8 | 55078876 | A/G | 0.623 | 0.584 | 1.11 | 0.025 | 3.30x10^-5^ |
| rs440007 | 8 | 86998242 | G/C | 0.317 | 0.277 | 1.11 | 0.027 | 8.70x10^-5^ |
| rs7873681 | 9 | 18428324 | A/G | 0.371 | 0.337 | 1.11 | 0.025 | 8.78x10^-5^ |
| rs9406749 | 9 | 18435976 | C/T | 0.479 | 0.444 | 1.10 | 0.025 | 7.33x10^-5^ |
| rs2791454 | 9 | 18436598 | C/T | 0.357 | 0.323 | 1.11 | 0.026 | 4.48x10^-5^ |
| rs9406750 | 9 | 18437473 | C/A | 0.356 | 0.323 | 1.11 | 0.026 | 5.08x10^-5^ |
| rs11789537 | 9 | 18438196 | T/C | 0.353 | 0.322 | 1.11 | 0.026 | 9.13x10^-5^ |
| rs2772399 | 9 | 18441146 | A/G | 0.356 | 0.324 | 1.11 | 0.026 | 9.30x10^-5^ |
| rs7043305 | 9 | 18441918 | T/C | 0.355 | 0.324 | 1.11 | 0.026 | 8.91x10^-5^ |
| rs2938883 | 9 | 81186382 | C/A | 0.097 | 0.077 | 1.22 | 0.044 | 9.59x10^-6^ |
| rs62576963 | 9 | 90809428 | G/A | 0.279 | 0.243 | 1.12 | 0.028 | 7.57x10^-5^ |
| rs11142158 | 9 | 90809598 | G/A | 0.279 | 0.243 | 1.12 | 0.028 | 7.46x10^-5^ |
| rs77922938 | 9 | 111060196 | A/G | 0.959 | 0.943 | 1.27 | 0.060 | 4.95x10^-5^ |
| rs10437535 | 10 | 805389 | C/T | 0.178 | 0.153 | 1.16 | 0.036 | 4.95x10^-5^ |
| rs112751353 | 10 | 2344595 | A/T | 0.074 | 0.011 | 3.89 | 0.345 | 8.31x10^-5^ |
| rs117852002 | 10 | 26560537 | A/G | 0.018 | 0.009 | 1.70 | 0.135 | 7.64x10^-5^ |
| rs184097363 | 10 | 35068410 | A/G | 0.011 | 0.005 | 5.58 | 0.434 | 7.51x10^-5^ |
| rs1807014 | 11 | 86493121 | C/T | 0.336 | 0.293 | 1.12 | 0.026 | 3.54x10^-5^ |
| rs7940637 | 11 | 86495697 | C/T | 0.378 | 0.335 | 1.12 | 0.026 | 1.52x10^-5^ |
| rs12809023 | 11 | 86495961 | C/G | 0.343 | 0.299 | 1.12 | 0.026 | 2.93x10^-5^ |
| rs6592315 | 11 | 86496427 | T/G | 0.340 | 0.297 | 1.12 | 0.026 | 2.54x10^-5^ |
| rs7123349 | 11 | 86496731 | G/T | 0.340 | 0.297 | 1.12 | 0.026 | 2.27x10^-5^ |
| rs11601553 | 11 | 86498414 | A/C | 0.360 | 0.317 | 1.11 | 0.026 | 2.85x10^-5^ |
| rs11602695 | 11 | 86500206 | A/G | 0.315 | 0.273 | 1.12 | 0.027 | 3.86x10^-5^ |
| rs17819134 | 11 | 86501339 | G/C | 0.315 | 0.272 | 1.12 | 0.027 | 3.34x10^-5^ |
| rs1939115 | 11 | 86504155 | T/A | 0.315 | 0.272 | 1.12 | 0.027 | 3.37x10^-5^ |
| rs12421942 | 11 | 86508487 | T/G | 0.314 | 0.272 | 1.11 | 0.027 | 5.62x10^-5^ |
| rs1939110 | 11 | 86515072 | C/T | 0.314 | 0.273 | 1.11 | 0.027 | 6.47x10^-5^ |
| rs114090928 | 11 | 106467718 | A/C | 0.980 | 0.968 | 1.42 | 0.090 | 9.30x10^-5^ |
| rs187141130 | 11 | 106509133 | A/G | 0.988 | 0.979 | 1.52 | 0.107 | 9.59x10^-5^ |
| rs72985385 | 11 | 106509638 | G/A | 0.992 | 0.981 | 1.62 | 0.122 | 8.64x10^-5^ |
| rs1382567372 | 12 | 95885908 | T/TTGTGTG | 0.543 | 0.509 | 1.11 | 0.027 | 5.49x10^-5^ |
| rs147790408 | 13 | 43818028 | T/C | 0.990 | 0.966 | 2.22 | 0.196 | 4.53x10^-5^ |
| rs373098847 | 13 | 77455805 | T/TA | 0.372 | 0.332 | 1.23 | 0.051 | 7.63x10^-5^ |
| rs12589308 | 14 | 34275727 | C/G | 0.125 | 0.105 | 1.18 | 0.039 | 3.99x10^-5^ |
| rs57454873 | 14 | 65702594 | T/G | 0.265 | 0.231 | 1.12 | 0.028 | 6.76x10^-5^ |
| rs61989807 | 14 | 65702941 | T/C | 0.263 | 0.229 | 1.12 | 0.028 | 8.95x10^-5^ |
| rs7142951 | 14 | 65704144 | T/C | 0.263 | 0.229 | 1.12 | 0.028 | 9.71x10^-5^ |
| rs7142218 | 14 | 65704433 | C/T | 0.263 | 0.229 | 1.12 | 0.028 | 9.38x10^-5^ |
| rs112753607 | 14 | 65708746 | C/CA | 0.213 | 0.188 | 1.15 | 0.033 | 3.46x10^-5^ |
| rs7146907 | 14 | 65717871 | C/T | 0.261 | 0.227 | 1.12 | 0.028 | 4.44x10^-5^ |
| rs59978001 | 14 | 65718674 | G/A | 0.263 | 0.230 | 1.12 | 0.028 | 8.37x10^-5^ |
| rs2086465 | 14 | 65719215 | G/T | 0.265 | 0.230 | 1.12 | 0.028 | 6.52x10^-5^ |
| rs8015540 | 14 | 65720065 | G/A | 0.264 | 0.230 | 1.12 | 0.028 | 6.28x10^-5^ |
| rs149115343 | 14 | 79963151 | A/G | 0.992 | 0.983 | 1.95 | 0.168 | 6.97x10^-5^ |
| 15:27464356 | 15 | 27464356 | A/G | 0.449 | 0.410 | 1.11 | 0.025 | 4.13x10^-5^ |
| rs77102660 | 15 | 57938396 | A/G | 0.993 | 0.977 | 3.27 | 0.281 | 2.43x10^-5^ |
| rs4624198 | 16 | 7458057 | C/T | 0.577 | 0.543 | 1.10 | 0.025 | 6.09x10^-5^ |
| rs200869063 | 16 | 60225631 | T/TAA | 0.784 | 0.756 | 1.13 | 0.031 | 8.21x10^-5^ |
| rs11865210 | 16 | 60227786 | T/C | 0.793 | 0.767 | 1.12 | 0.030 | 9.55x10^-5^ |
| rs117171470 | 17 | 50381798 | A/G | 0.977 | 0.967 | 1.36 | 0.079 | 9.23x10^-5^ |
| rs12605882 | 18 | 71021844 | C/T | 0.124 | 0.104 | 1.14 | 0.031 | 4.09x10^-5^ |
| rs78161503 | 18 | 71024040 | G/A | 0.124 | 0.105 | 1.13 | 0.031 | 5.81x10^-5^ |
| rs1943794 | 18 | 71024963 | G/A | 0.124 | 0.105 | 1.13 | 0.031 | 6.32x10^-5^ |
| rs142459400 | 18 | 71025453 | TA/T | 0.124 | 0.106 | 1.13 | 0.031 | 7.67x10^-5^ |
| rs547522376 | 18 | 71025487 | A/ATT | 0.124 | 0.106 | 1.13 | 0.031 | 7.12x10^-5^ |
| rs28619385 | 18 | 71026383 | G/A | 0.124 | 0.105 | 1.13 | 0.031 | 7.43x10^-5^ |
| rs76179356 | 18 | 71026922 | G/A | 0.125 | 0.105 | 1.13 | 0.031 | 5.51x10^-5^ |
| rs1943797 | 18 | 71027518 | A/C | 0.124 | 0.105 | 1.13 | 0.031 | 5.89x10^-5^ |
| rs77817048 | 18 | 71030136 | G/A | 0.124 | 0.105 | 1.13 | 0.031 | 6.19x10^-5^ |
| rs140244004 | 18 | 71030699 | AAGG/A | 0.124 | 0.104 | 1.14 | 0.032 | 4.26x10^-5^ |
| rs35521455 | 18 | 71030895 | G/A | 0.124 | 0.105 | 1.14 | 0.031 | 5.94x10^-5^ |
| rs35731318 | 18 | 71031039 | G/A | 0.124 | 0.105 | 1.13 | 0.031 | 6.10x10^-5^ |
| rs138045934 | 18 | 71034657 | C/T | 0.127 | 0.109 | 1.13 | 0.032 | 8.70x10^-5^ |
| rs76025710 | 18 | 71038706 | G/C | 0.126 | 0.109 | 1.15 | 0.033 | 3.68x10^-5^ |
| rs747866565 | 21 | 36972001 | A/AGAG | 0.417 | 0.368 | 1.11 | 0.027 | 9.36x10^-5^ |
| rs12329908 | 21 | 36976115 | C/T | 0.376 | 0.340 | 1.11 | 0.026 | 8.34x10^-5^ |
| rs5843721 | 21 | 36978462 | A/AAGG | 0.378 | 0.341 | 1.11 | 0.026 | 8.27x10^-5^ |
| rs2222960 | 21 | 36978646 | C/T | 0.378 | 0.341 | 1.11 | 0.025 | 7.97x10^-5^ |
| rs2835033 | 21 | 36978966 | T/C | 0.378 | 0.341 | 1.11 | 0.025 | 7.78x10^-5^ |
| rs2835034 | 21 | 36979082 | C/G | 0.378 | 0.341 | 1.11 | 0.025 | 7.65x10^-5^ |
| rs9982956 | 21 | 36979285 | G/A | 0.379 | 0.341 | 1.11 | 0.025 | 6.54x10^-5^ |
| rs9305568 | 21 | 36979855 | T/A | 0.378 | 0.341 | 1.11 | 0.025 | 6.49x10^-5^ |
| rs9647069 | 21 | 36980135 | T/C | 0.378 | 0.341 | 1.11 | 0.025 | 6.38x10^-5^ |
| rs9305569 | 21 | 36981259 | T/C | 0.376 | 0.340 | 1.11 | 0.025 | 5.67x10^-5^ |
| rs2835037 | 21 | 36984024 | C/T | 0.378 | 0.341 | 1.11 | 0.025 | 6.93x10^-5^ |
| rs28736599 | 21 | 36984094 | A/G | 0.378 | 0.341 | 1.11 | 0.025 | 6.70x10^-5^ |
| rs9982035 | 21 | 36984290 | G/A | 0.379 | 0.341 | 1.11 | 0.026 | 7.56x10^-5^ |
| rs9982040 | 21 | 36984295 | G/T | 0.379 | 0.341 | 1.11 | 0.026 | 7.56x10^-5^ |
| rs9984647 | 21 | 36984955 | C/T | 0.378 | 0.341 | 1.11 | 0.025 | 6.70x10^-5^ |
| rs9984738 | 21 | 36985109 | A/G | 0.378 | 0.340 | 1.11 | 0.025 | 6.59x10^-5^ |
| rs796695391 | 21 | 36985144 | AC/A | 0.378 | 0.341 | 1.11 | 0.026 | 7.75x10^-5^ |
| rs7277603 | 21 | 36985167 | C/T | 0.378 | 0.340 | 1.11 | 0.025 | 6.59x10^-5^ |
| rs2835039 | 21 | 36985190 | A/G | 0.378 | 0.341 | 1.11 | 0.025 | 6.59x10^-5^ |
| rs2835040 | 21 | 36985345 | A/G | 0.378 | 0.341 | 1.11 | 0.025 | 6.43x10^-5^ |
| rs2142034 | 21 | 36990175 | A/T | 0.375 | 0.339 | 1.11 | 0.025 | 7.15x10^-5^ |
| rs10222105 | 21 | 36991949 | A/G | 0.377 | 0.340 | 1.11 | 0.025 | 7.10x10^-5^ |
| rs73203438 | 21 | 36996758 | C/A | 0.361 | 0.325 | 1.11 | 0.026 | 5.61x10^-5^ |
| rs9979464 | 21 | 37000860 | G/A | 0.378 | 0.340 | 1.11 | 0.026 | 5.71x10^-5^ |
| rs2835050 | 21 | 37001464 | A/G | 0.375 | 0.339 | 1.11 | 0.026 | 5.66x10^-5^ |
| rs2835060 | 21 | 37009121 | A/T | 0.378 | 0.340 | 1.11 | 0.026 | 4.91x10^-5^ |
| rs2835061 | 21 | 37009507 | G/A | 0.378 | 0.340 | 1.11 | 0.025 | 5.21x10^-5^ |
| rs2835062 | 21 | 37010309 | G/A | 0.378 | 0.340 | 1.11 | 0.026 | 5.17x10^-5^ |
| rs2835063 | 21 | 37013060 | C/T | 0.375 | 0.338 | 1.11 | 0.026 | 5.00x10^-5^ |
| rs61229183 | 21 | 37017335 | AT/A | 0.367 | 0.328 | 1.11 | 0.026 | 4.98x10^-5^ |
| rs2242761 | 21 | 37024845 | G/A | 0.365 | 0.327 | 1.11 | 0.026 | 2.95x10^-5^ |
| rs62219101 | 21 | 37048377 | G/A | 0.372 | 0.334 | 1.11 | 0.026 | 4.56x10^-5^ |
| rs55985814 | 21 | 37052768 | G/C | 0.371 | 0.333 | 1.11 | 0.026 | 3.51x10^-5^ |
| rs737407 | 21 | 37054070 | C/T | 0.370 | 0.332 | 1.11 | 0.026 | 3.31x10^-5^ |
| rs737408 | 21 | 37054325 | C/A | 0.370 | 0.331 | 1.11 | 0.026 | 3.07x10^-5^ |
| rs737406 | 21 | 37054396 | C/G | 0.370 | 0.331 | 1.11 | 0.026 | 3.15x10^-5^ |
| rs4995476 | 21 | 37054785 | C/T | 0.369 | 0.331 | 1.11 | 0.026 | 3.37x10^-5^ |
| rs11911644 | 21 | 37054924 | C/T | 0.370 | 0.331 | 1.11 | 0.026 | 3.12x10^-5^ |
| rs11911676 | 21 | 37055083 | C/G | 0.370 | 0.331 | 1.11 | 0.026 | 3.07x10^-5^ |
| rs2835083 | 21 | 37055140 | G/C | 0.372 | 0.333 | 1.11 | 0.026 | 3.02x10^-5^ |
| rs2242764 | 21 | 37058879 | C/T | 0.373 | 0.334 | 1.11 | 0.026 | 3.34x10^-5^ |
| rs11088325 | 21 | 37058995 | C/G | 0.375 | 0.339 | 1.11 | 0.026 | 6.08x10^-5^ |
| rs11088327 | 21 | 37059234 | A/G | 0.376 | 0.337 | 1.11 | 0.026 | 3.85x10^-5^ |
| rs9305684 | 21 | 41334298 | C/T | 0.576 | 0.542 | 1.10 | 0.025 | 9.56x10^-5^ |

**Supplementary Table 2: Fisher’s Exact Tests**

|  |  | **Overlapping Chondrogenic Dif.** | **Not Overlapping Chondrogenic Dif.** |  |  | **Overlapping NH Osteoblasts** | **Not Overlapping NH Osteoblasts** | *Row total* |
| --- | --- | --- | --- | --- | --- | --- | --- | --- |
|  | **p<1x10^-4^** | 30 | 23 |  |  | 29 | 24 | *53* |
|  | **p≥1x10^-4^** | 583,653 | 1,384,515 |  |  | 614,948 | 1,353,220 | *1,968,168* |
|  | *Column total* | *583,673* | *1,384,538* |  |  | *614,977* | *1,353,244* | *1,968,221* |
|  |  | **p-value:** 3.99x10^-5^  **odds ratio:** 3.09 | |  |  | **p-value:** 3.33x10^-4^  **odds ratio:** 2.66 | | |

The counts in the above table represent the number of markers outside the MHC (26Mbp-34Mbp) from psoriasis PsA/PsC indirect meta-analysis across 6 cohorts, that overlap active enhancers for at least one cell type
